# Supplementary material for: Meta-Analysis and Gene Set Enrichment Relative to ER Status Reveal Elevated Activity of MYC and E2F in the “Basal” Breast Cancer Subgroup
Source: PLoS One. 2009 Mar 9;4(3):e4710. doi: 10.1371/journal.pone.0004710 (PMC2650420; doi:10.1371/journal.pone.0004710)
Supplement: Dataset S1 — GSEA results from first screen. The file contains a zipped archive of the HTML results of the second GSEA screen. Reports for enriched or depleted datasets may be accessed from the “index.html” link within the my_analysis.GseaPreranked.1219975950625.rpt.zip ZIP archive. Enrichment plots are available for each of the 50 most enriched and depleted gene sets. (13.32 MB ZIP) [file pone.0004710.s001.zip › my_analysis.GseaPreranked.1219975950625.rpt.zip!//CROONQUIST_IL6_RAS_DN.html]

Details for gene set CROONQUIST\_IL6\_RAS\_DN[GSEA]

|  || Dataset | zSco.ERneg.to.ERpos\_collapsed |
| Phenotype | NoPhenotypeAvailable |
| Upregulated in class | na\_pos |
| GeneSet | CROONQUIST\_IL6\_RAS\_DN |
| Enrichment Score (ES) | 0.7423936 |
| Normalized Enrichment Score (NES) | 2.3152235 |
| Nominal p-value | 0.0 |
| FDR q-value | 0.0 |
| FWER p-Value | 0.0 |
Table: GSEA Results Summary

  

Fig 1: Enrichment plot: CROONQUIST\_IL6\_RAS\_DN      
 Profile of the Running ES Score & Positions of GeneSet Members on the Rank Ordered List

  

| PROBE | GENE SYMBOL | GENE\_TITLE | RANK IN GENE LIST | RANK METRIC SCORE | RUNNING ES | CORE ENRICHMENT || 1 | CDC20 |  |  | 9 | 7.151 | 0.0756 | Yes |
| 2 | FOXM1 |  |  | 21 | 6.760 | 0.1469 | Yes |
| 3 | CENPA |  |  | 51 | 6.315 | 0.2121 | Yes |
| 4 | MKI67 |  |  | 98 | 5.871 | 0.2713 | Yes |
| 5 | KIF2C |  |  | 124 | 5.703 | 0.3303 | Yes |
| 6 | EXO1 |  |  | 218 | 5.239 | 0.3792 | Yes |
| 7 | BUB1B |  |  | 271 | 5.016 | 0.4288 | Yes |
| 8 | OIP5 |  |  | 320 | 4.868 | 0.4771 | Yes |
| 9 | MYBL2 |  |  | 418 | 4.629 | 0.5192 | Yes |
| 10 | CCNB2 |  |  | 431 | 4.589 | 0.5673 | Yes |
| 11 | SPBC25 |  |  | 606 | 4.126 | 0.5982 | Yes |
| 12 | MCM3 |  |  | 656 | 4.019 | 0.6374 | Yes |
| 13 | UBE2C |  |  | 994 | 3.489 | 0.6493 | Yes |
| 14 | CDC2 |  |  | 1259 | 3.182 | 0.6634 | Yes |
| 15 | POLD1 |  |  | 1260 | 3.182 | 0.6973 | Yes |
| 16 | CCNB1 |  |  | 1626 | 2.823 | 0.7000 | Yes |
| 17 | CDC25C |  |  | 1794 | 2.700 | 0.7163 | Yes |
| 18 | CDKN3 |  |  | 1827 | 2.674 | 0.7424 | Yes |
| 19 | SBNO2 |  |  | 2402 | 2.260 | 0.7233 | No |
| 20 | TRAIP |  |  | 3007 | 1.928 | 0.6985 | No |
| 21 | WEE1 |  |  | 3048 | 1.909 | 0.7158 | No |
| 22 | CDC6 |  |  | 3185 | 1.847 | 0.7253 | No |
| 23 | KIF22 |  |  | 8725 | -0.180 | 0.3107 | No |
| 24 | GALE |  |  | 12501 | -3.262 | 0.0616 | No |
Table: GSEA details [plain text format]

  

Fig 2: CROONQUIST\_IL6\_RAS\_DN: Random ES distribution      
 Gene set null distribution of ES for **CROONQUIST\_IL6\_RAS\_DN**
